# High Fat Diet Induces Formation of Spontaneous Liposarcoma in Mouse Adipose Tissue with Overexpression of Interleukin 22

**DOI:** 10.1371/journal.pone.0023737

**Published:** 2011-08-29

**Authors:** Zheng Wang, Ling Yang, Yuhui Jiang, Zhi-Qiang Ling, Zhigang Li, Yuan Cheng, Heng Huang, Lingdi Wang, Yi Pan, Zhenzhen Wang, Xiaoqiang Yan, Yan Chen

**Affiliations:** 1 Key Laboratory of Nutrition and Metabolism, Institute for Nutritional Sciences, Shanghai Institutes for Biological Sciences, Chinese Academy of Sciences, Graduate School of the Chinese Academy of Sciences, Shanghai, China; 2 Zhejiang Cancer Research Institute, Zhejiang Province Cancer Hospital, Zhejiang Cancer Center, Hangzhou, China; 3 Generon Corporation, Zhang Jiang Hi-Tech Park, Shanghai, China; University of Hong Kong, China

## Abstract

Interleukin 22 (IL-22) is a T-cell secreted cytokine that modulates inflammatory response in nonhematopoietic tissues such as epithelium and liver. The function of IL-22 in adipose tissue is currently unknown. We generated a transgenic mouse model with overexpression of IL-22 specifically in adipose tissue. The IL-22 transgenic mice had no apparent changes in obesity and insulin resistance after feeding with high fat diet (HFD). Unexpectedly, all the IL-22 transgenic mice fed with HFD for four months developed spontaneous tumors in epididymal adipose tissue. Histological analysis indicated that the tumors were well-differentiated liposarcomas with infiltration of inflammatory cells. IL-22 overexpression promotes production of inflammatory cytokines such as IL-1β and IL-10 and stimulates ERK phosphorylation in adipose tissue. Furthermore, IL-22 treatment in differentiated 3T3-L1 adipocytes could induce IL-1β and IL-10 expression, together with stimulation of ERK phosphorylation. Taken together, our study not only established a novel mouse model with spontaneous liposarcoma, but also revealed that IL-22 overexpression may collaborate with diet-induced obesity to impact on tumor development in mouse.

## Introduction

Interleukin (IL)-22 is a T cell secreted and IL-10-related cytokine that was first identified in 2000 [1], [2]. IL-22 exerts its biological activities on tissues such as skin, liver, kidney, respiratory and digestive systems, but not on cells of hematopoietic lineage. The effect of IL-22 as a cytokine is dependent on the location of its action as well as the local cytokine milieu [3], [4]. For instance, IL-22 can exert either pro-inflammatory or anti-inflammatory effect dependent on cellular context [3], [4]. IL-22 has a pro-inflammatory effect in a mouse model of psoriasis-like skin inflammation [5]. It also has a pro-inflammatory effect in promoting bacterial load and organ failure during septic peritonitis [6]. On the other hand, IL-22 has an anti-inflammatory effect to ameliorate local intestinal inflammation in a mouse model of ulcerative colitis [7]. A fusion gene between IL-22 and Ig ameliorates experimental autoimmune myocarditis in rats [8]. IL-22 also has an *in vivo* protective function in mice against concanavalin A-, carbon tetrachloride-, and Fas ligand-induced hepatic injury, necrosis and apoptosis [9]. In addition, IL-22 has a protective function in alcohol-induced liver injury [10], as well as T cell-mediated hepatitis in the mouse [11]. Consistently, overexpression of IL-22 in mouse liver can ameliorate concanavalin A-induced T cell hepatitis [12].

In addition to its primary role in modulation of inflammation, IL-22 contributes to tumor cell growth and apoptosis. It was reported that IL-22 can promote cell growth and survival in HepG2 cells by activating STAT3 and inducing expression of a variety of anti-apoptotic and mitogenic proteins [11]. In non-small cell lung carcinoma (NSCLC), overexpression of IL-22 protected lung cancer cell lines from apoptosis, while downregulation of IL-22 significantly inhibited the human tumor cell growth in BALB/c nude mice [13]. In contrast, in breast cancer cells, IL-22 was found to effectively reduce the growth of tumor cells, correlated with an inhibition on ERK and AKT phosphorylation and induction of cell cycle arrest [14]. Therefore, the effect of IL-22 on cancer development appears to be dependent on cellular context.

Our group recently found that IL-22 play a protective role in high fat diet induced liver steatosis through down-regulating the expression of lipogenesis-related genes including critical transcription factors and enzymes for lipid synthesis [15]. In this study, we further investigated the potential function of IL-22 on adipose tissue by generation of a transgenic mouse model with adipose-specific expression of IL-22. Interestingly, the mice with overexpression of IL-22 in adipose tissue had neither apparent phenotype nor metabolic alteration when fed with high fat diet. However, the IL-22 transgenic mice developed spontaneous liposarcomas in adipose tissue after long-term feeding with high fat diet, indicating that diet may interact with inflammation changes associated with IL-22 overexpression in tumorigenesis in adipose tissue.

## Materials and Methods

### Generation of IL-22 transgenic mouse

All animals were kept and used under the guidelines of the Institutional Animal Care and Use Committee of the Institute for Nutritional Sciences, Chinese Academy of Sciences (CAS), with free access to standard mouse chow and tap water. All of the experimental procedures were carried out in accordance with the CAS ethics commission with an approval number 2010-AN-8. The full length mouse IL-22 cDNA was amplified by RT-PCR with mouse thymus cDNA. After confirmation by DNA sequencing, the mouse cDNA was cloned into pBS-aP2-sv40pA vector (from Addgene, Cambridge, MA, USA). To generate transgenic mice, the transgenic cassette was excised from the plasmid and used in microinjection into the pronuclei of fertilized oocytes of the ICR strain of mice. The transgenic mice were genotyped by PCR with genomic DNA with primers 5′-AAACATACAGGGTCTGGTCAT-3′ and 5′-GCATAAAGGTGCGGTTGA -3′. All the mice used in this study were of ICR background.

### Genomic DNA extraction, RNA isolation, reverse transcription and PCR (RT-PCR), and quantitative real-time RT-PCR (qRT-PCR)

Genomic DNA was extracted from mouse tail with heating at 100°C for at least 1 hour in 50 mM NaOH, followed by addition of Tris-Cl solution (pH 8.0). Total RNA was extracted from tissues of the mice or cells using Trizol reagent (Invitrogen, Carlsbad, CA, USA) according to the manufacturer's protocol. Reverse transcription-PCR (RT-PCR) was performed as previously described [15]. The primers for RT-PCR were as follows: 5′-GTGGGATCCCTGATGGCTGTCCTGCAG-3′ and 5′-AGCGAATTCTCGCTCAGACTGCAAGCAT-3′ for mouse IL-22, 5′-ATGAAGACACTACTGACCATCCT-3′ and 5′- CAGCCACTTTCTCTCTCCGT -3′ for mouse IL-22R1, 5′- AGGGCTGAATTTGCAGATGA -3′ and 5′- CCGTTTTTCCAGTATTGCAC-3′ for mouse IL-10R2, 5′-CAACGGCACAGTCAAGG-3′ and 5′-AAGTCGCAGGAGACAACC-3′ for mouse GAPDH. The expression levels of IL-22, IL-1β, IL-10, and IFN-γ were determined by qRT-PCR using SYBR Green real-time PCR master mix (TOYOBO, Osaka, Japan) with 7900 real-time instrument (Applied Biosystems, Carlsbad, CA, USA). The primers used are as follows for qRT-PCR: 5′-CATGCAGGAGGTGGTACCTT -3′ and 5′-CAGACGCAAGCATTTCTCAG-3′ for mouse IL-22, 5′-AAAAAAGCCTCGTGCTGTCG-3′ and 5′-GTCGTTGCTTGGTTCTCCTTG -3′ for mouse IL-1β, 5′- ACTGCACCCACTTCCCAGT-3′ and 5′-TTGTCCAGCTGGTCCTTTGT-3′ for mouse IL-10, 5′-TAGCCAAGACTGTGATTGCGG-3′ and 5′-AGACATCTCCTCCCATCAGCAG-3′ for mouse IFN-γ, 5′- TCCATCCAGTTGCCTTCTTG-3′ and 5′-TTCCACGATTTCCCAGAGAAC-3′ for mouse IL-6, 5′-AAGCCTGTAGCCCACGTCGTA-3′ and 5′-AGGTACAACCCATCGGCTGG -3′ for mouse TNF-α, 5′-GATCATTGCTCCTCCTGAGC-3′ and 5′-ACTCCTGCTTGCTGATCCAC-3′ for β-actin used to normalize qRT-PCR.

### Analysis of food intake, glucose tolerance test (GTT), and insulin tolerance test (ITT)

The male wild type and IL-22 transgenic male mice of 1 month old were fed with either normal chow (SLACOM, Shanghai, China) or high fat diet (containing 60% calories from fat, from Research Diets Inc. New Brunswick, NJ, USA). Food intake was recorded every two days. For GTT and ITT, the mice were fasted overnight, followed by intraperitoneal injection of glucose (1 g/kg body weight, for GTT) or insulin (1 U/kg body weight, for ITT) respectively. Blood glucose levels at various times after the injection were determined by an electronic glucometer (Freestyle Freedom, Abbott Diabetes Care, Alameda, CA, USA).

### Hematoxylin-eosin staining and immunohistochemistry

Fresh epididymal white adipose tissue (eWAT) and tumors were fixed in 10% formalin/phosphate-buffered saline, and then embedded with paraffin. Sections were subjected to standard hematoxylin-eosin (HE) staining and immunohistochemistry as previously described [16].

### 3T3-L1 cell culture, differentiation, IL-22 treatment, and immunoblotting

3T3-L1 mouse fibroblast cells were obtained from the Bank/Stem Cell Bank, Shanghai Institute for Biological Sciences, Chinese Academy of Sciences. The cells were grown under 5% CO_2_ in standard medium containing Dulbecco's Modified Eagles Medium (GIBCO,Carlsbad, CA, USA), 10% fetal bovine serum (FBS) and 0.1% penicillin-streptomycin mixture (GIBCO). For adipocyte differentiation, 3T3-L1 cells were allowed to reach confluence and at two days post confluence (day 0), the cells were induced to differentiate under 10% CO_2_ environments with a medium containing 10% fetal bovine serum, 10 µg/ml insulin, 1 µM dexamethasone, and 0.5 mM IBMX for 2 days. Thereafter, post-differentiation medium containing only insulin and 10% FBS was added and the cells were cultured for 2 more days. From then on the culture medium was replenished every 2 days with standard medium. Fully differentiated 3T3-L1 cells were treated for 48 hours with 500 ng/ml recombinant IL-22 as described previously [15]. The IL-22-treated cells were then used for RT-PCR and immunoblotting as previously described [15]. Phospho-ERK1/2 antibody was purchased from Cell Signaling Technology (Danvers, MA, USA). Total ERK1/2 antibody was from Santa Cruz Biotechnology (Santa Cruz, CA, USA). The antibody for tubulin was from Sigma-Aldrich (St. Louis, MO, USA).

### Measurement of serum IL-22 concentration

The protein concentration of mouse serum IL-22 was measured by an ELISA kit from eBioscience (San Diego, CA, USA) following manufacturer's instruction.

### Statistical analysis

All the data were analyzed by unpaired two-tailed Student's t-test and expressed as means ± standard deviation.

## Results

### Generation and characterization of transgenic mice with IL-22 expression in adipose tissues

**Figure 1:**
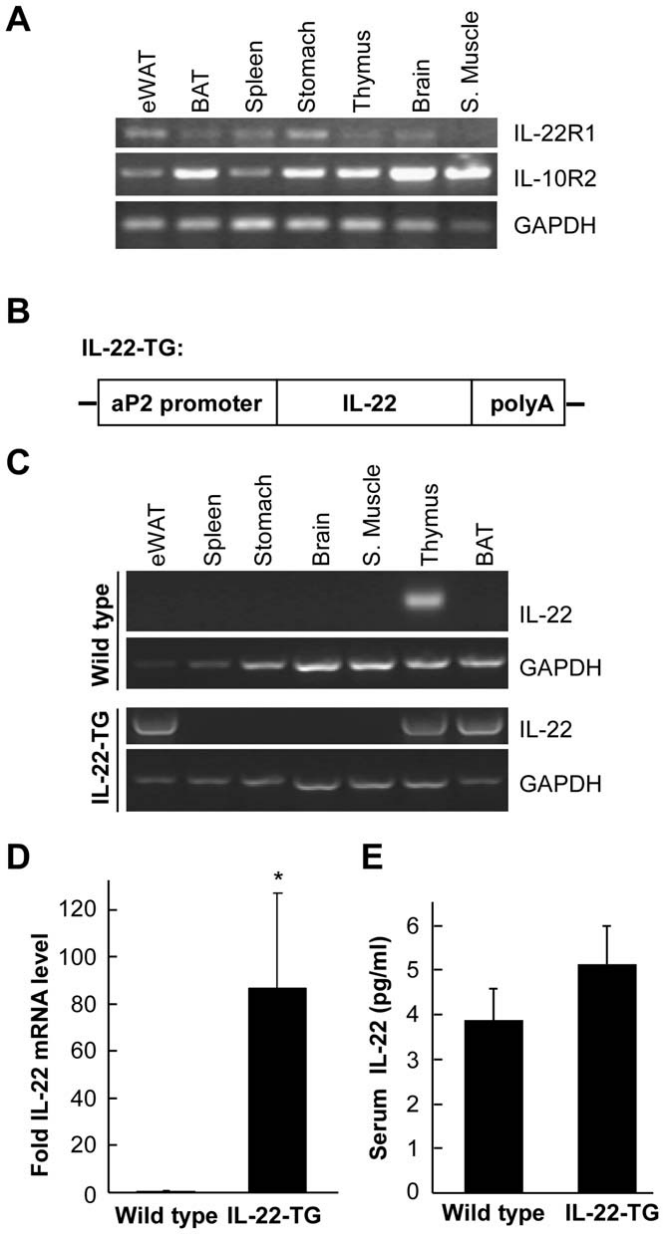
Generation of transgenic mice with IL-22 specifically expressed in adipose tissue. (A) IL-22 receptors are present in adipose tissues. Various tissues were isolated from ICR mice and used in RT-PCR to detect the expression levels of IL-22R1 and IL-10R2. GAPDH was used as a loading control. eWAT stands for epididymal white fat and BAT for brown fat. (B) A diagram to depict the transgenic construct. The full length mouse IL-22 cDNA was cloned downstream of aP2 promoter. (C) Analysis of IL-22 mRNA levels in wild type and IL-22 transgenic (IL-22-TG) mice. Various mouse tissues were used in RT-PCR to detect mRNA level of IL-22. Note that in comparison with wild type control, IL-22 expression was markedly increased in eWAT and BAT in IL-22-TG mice. (D) Analysis of IL-22 level of mouse eWAT by real-time quantitative RT-PCR (qRT-PCR). The relative expression level of IL-22 compared with β-actin was shown as mean ± SD (n = 7 for each group). * indicates p<0.05 between the two groups. (E) Measurement of serum IL-22 protein levels. The sera of both wild type and IL-22-TG mice were used to determine the IL-22 protein level. The data are shown as mean ± SD (n = 7 for each group).

Our previous study has indicated that IL-22 has an effect on lipogenesis in the liver [15]. We therefore speculated that IL-22 might possess a function in adipose tissue. IL-22 exerts its effect by interacting with two membrane receptors, a specific receptor IL-22R1 and a common component IL-10R2 [17]. We analyzed whether adipose tissues express these two receptors. Different mouse tissues were used to isolate total RNA, followed by analysis with RT-PCR (Figure 1A). We found that both of the IL-22 receptors were present in many mouse tissues including epididymal white adipose tissue (eWAT) and brown fat tissue (BAT), indicating that IL-22 may play a functional role in these tissues.

To further analyze the potential function of IL-22 on adipose tissues, we generated a transgenic mouse model with IL-22 overexpression in adipocytes. The full-length mouse IL-22 cDNA was cloned downstream of aP2 promoter that specifies gene expression only in adipocytes (Figure 1B). The transgenic cassette was microinjected into the pronuclei of fertilized ICR mouse eggs to generate IL-22 transgenic (IL-22-TG) mice. IL-22-TG mice were then crossed with ICR mice to generate offspring used in the study. In wild type mice, IL-22 mRNA was mainly present in the thymus (Figure 1C). However, in IL-22-TG mice, IL-22 was highly expressed in eWAT and BAT (Figure 1C), confirming a successful overexpression of IL-22 in adipose tissues under aP2 promoter in the transgenic mice. We further analyzed the expression of IL-22 in eWAT using real-time quantitative RT-PCR method. We found that the IL-22 mRNA level in IL-22-TG mice was markedly elevated in comparison with the wild type animals (Figure 1D). However, the blood IL-22 protein level appeared not significantly affected by IL-22 overexpression in the adipose tissues.

### IL-22 overexpression in adipose tissue has no apparent effect on metabolism

**Figure 2:**
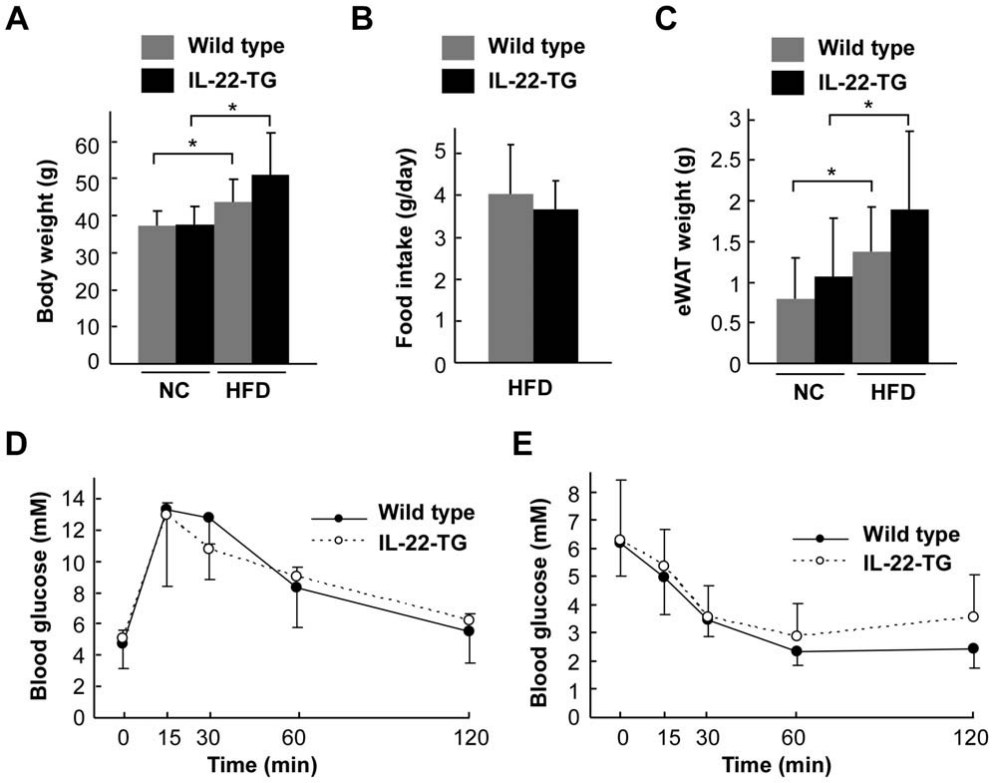
Metabolic profiles of wild type and IL-22-TG mice. (A to C) Body weight, food intake and epididymal white fat (eWAT) weight of wild type and IL-22-TG mice fed with either normal chow (NC) or high fat diet (HFD) for 4 months (n = 7 and 8 for the two groups respectively). (D, E) Glucose tolerance test (D) and insulin tolerance test (E) were performed with wild-type and IL-22-TG mice after fed with HFD for 4 months (n = 7 and 8 for the two groups respectively).

As adipose tissue is one of the major organs involved in the regulation of metabolism, we investigated the potential effect of IL-22 overexpression in adipose tissue on obesity and insulin resistance. The wild type and IL-22-TG male mice were fed with either normal chow (NC) or high fat diet (HFD) starting from age of 1-month. We analyzed their metabolic features after feeding with NC or HFD for 4 months. We found that HFD could increase the body weight in both wild type and IL-22-TG mice, but there was no significant difference in body weight between the two groups of mice under either NC or HFD (Figure 2A). The food intake was also not changed between the wild type and IL-22-TG mice fed with HFD for 4 months (Figure 2B). In addition, while HFD could elevate the weight of epididymal WAT, there was no significant difference between the two groups of mice (Figure 2C), indicating that IL-22 overexpression in adipose tissue has no significant effect on HFD-induced obesity. We next analyzed the potential effect of IL-22 overexpression in adipose tissue on glucose metabolism. By glucose tolerance test (GTT) and insulin tolerance test (ITT), we did not detect a significant difference between the two groups of mice fed after feeding with HFD (Figure 2D and 2E). Collectively, these data indicate that IL-22 overexpression has no apparent effect on HFD-induced obesity and insulin resistance *in vivo*.

### High fat diet induced formation of spontaneous liposarcomas in IL-22-TG mice

**Figure 3:**
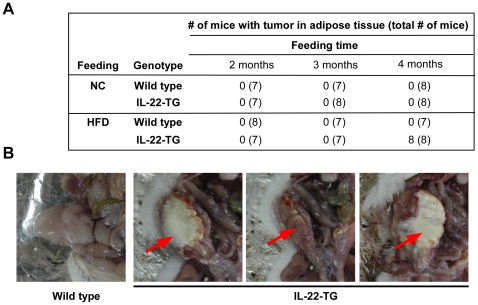
High fat diet induced spontaneous tumors in adipose tissue in IL-22-TG mice. (A) Summary of spontaneous tumor incidence in epididymal adipose tissue in mice fed with normal chow (NC) or high fat diet (HFD) for various length of time. HFD was started in the mice at 1-month-age. Note that spontaneous tumor formation in 100% of IL-22-TG mice after feeding with HFD for 4 months. (B) Representative pictures of epididymis of the wild type and IL-22-TG mice. The arrow indicates spontaneous tumors formed in the IL-22-TG mice fed with HFD for 4 months.

Unexpectedly, we found that long-term HFD feeding was able to induce formation of spontaneous tumors in adipose tissue. Both wild type and IL-22-TG mice were fed with either normal chow or HFD for 4 months. Surprisingly, 100% of IL-22-TG mice fed with HFD developed spontaneous tumors in epididymal adipose tissue (Figure 3A). However, none of the wild type mice fed with HFD or IL-22-TG mice fed with normal chow had tumors in adipose tissue (Figure 3A). All the tumors formed in the mice were within epididymis with most of them having necrosis in the middle (Figure 3B). We did not observe metastasis of the tumor to other organs in the mice (data not shown).

**Figure 4:**
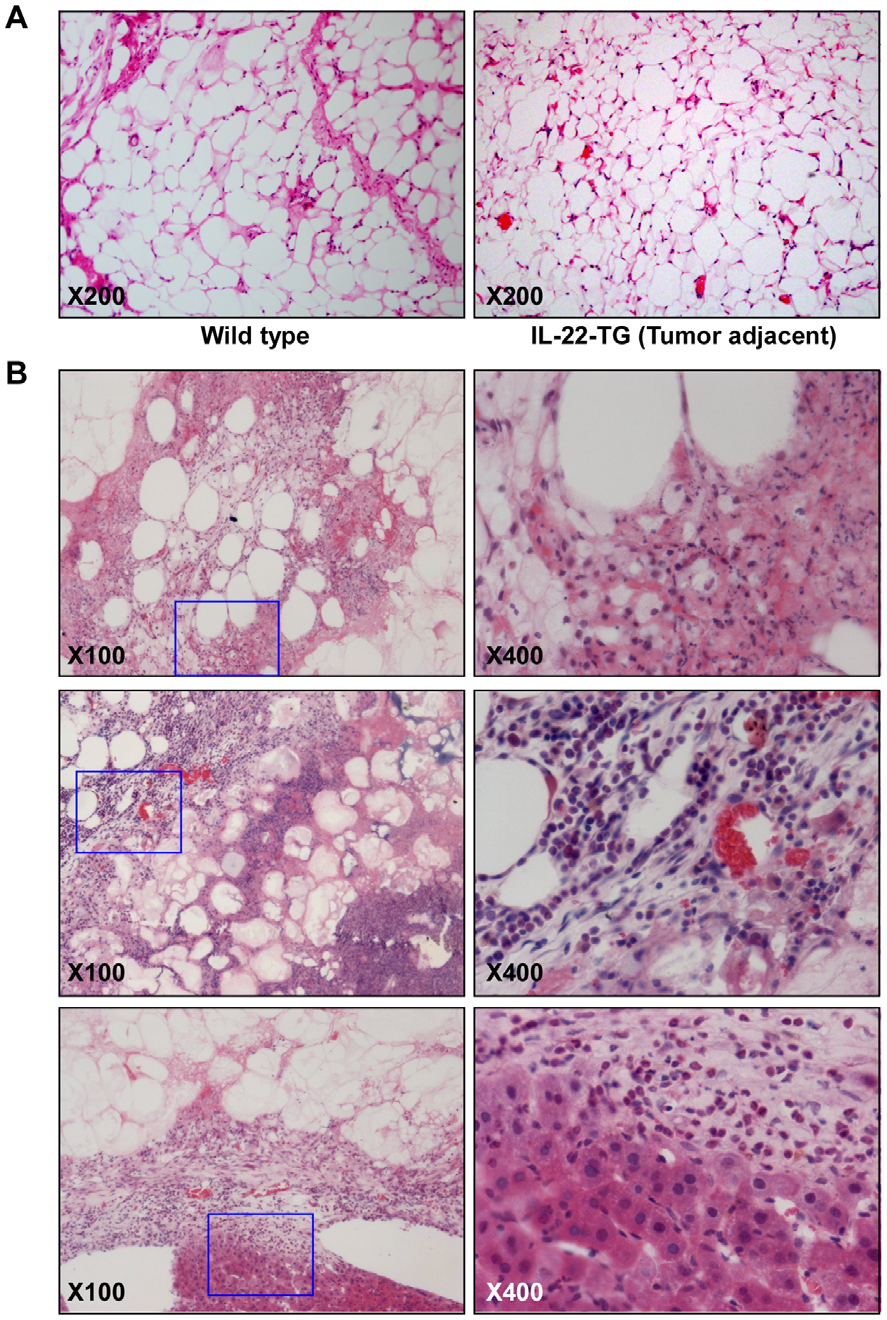
Histological and histochemical analyses of the liposarcoma in IL-22-TG mice fed with high fat diet. (A) HE staining of epididymal adipose tissue in wild type mouse and tumor adjacent tissue in IL-22-TG mouse. (B) HE staining of liposarcoma samples in IL-22-TG mice. The pictures in the right panel are amplified images of the inset inside the pictures in left panel (marked by blue solid line).

Histological analysis with hematoxylin-eosin (HE) staining revealed that the adipose tissue adjacent to the tumor were similar to that of wild type (Figure 4A). However, the shape of the adipocytes in IL-22-TG mice was not as regular as that of adipocytes in wild type mice (Figure 4A). HE staining with the tumor samples revealed that these tumors were most likely liposarcomas of the well-differentiated type (Figure 4B). Microscopically, the tumor was composed of broad sheets and streaks of adipocytes admixed with occasional lipoblasts, separated by fibrous septa containing spindle cells with hyperchromatic and mildly pleomorphic nuclei. Signet-ring cells resembling normal adipose tissue and multivacuolar lipoblasts were also seen. The size of the fat cells was variable and some lesions within the tumor were infiltrated by a small to moderate number of chronic inflammatory cells. Large nuclei and minor atypia of the cells were also observed. Therefore, a diagnosis of well-differentiated “lipoma-like” liposarcoma was made based on the histopathological appearance of the tumors (Figure 4B).

### IL-22 promotes production of inflammatory cytokines and ERK phosphorylation in adipose tissue and adipocytes

**Figure 5:**
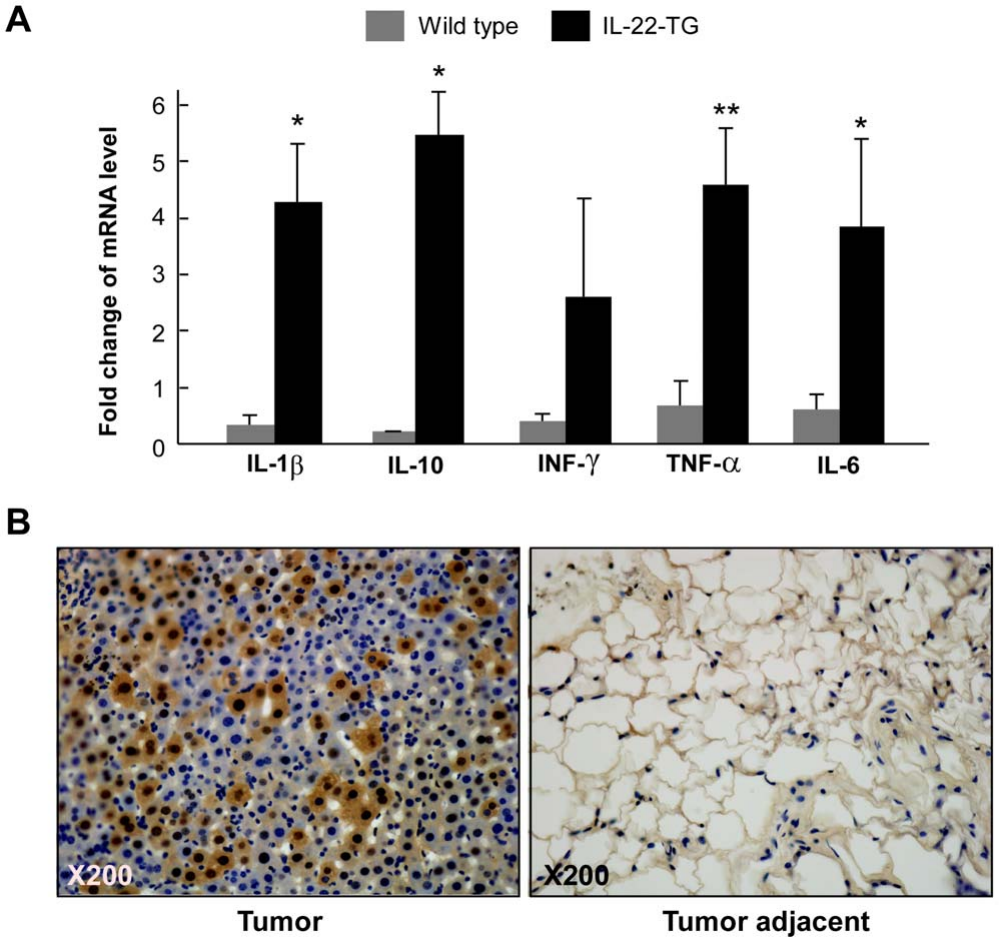
IL-22 promotes cytokine production and ERK phosphorylation in adipose tissue. (A) The mRNA levels of a set of inflammatory cytokines were increased in IL-22-TG mice. Epididymal white fat tissues were isolated from wild type and IL-22-TG mice (male, at 4 months old) and used in real-time quantitative RT-PCR to detect the expression levels of IL-1β, IL-10, INF-γ, TNF-α, and IL-6. The data are shown as mean ± SD (n = 7 for each group). * indicates p<0.05 and ** for p<0.01 between the two mouse groups. (B) Immunohistochemistry of liposarcoma and tumor-adjacent tissues to detect the level of phosphorylated ERK.

It has been proposed that inflammation contributes to tumorigenesis [18], and IL-22 has a functional role in modulating inflammatory response in peripheral tissues [3], [4]. We hypothesized that IL-22-mediated inflammatory response might, at least partially, contribute to the development of the tumors in adipose tissue. To address this hypothesis, we analyzed the effect of IL-22 overexpression on the expression of a set of inflammation-related cytokines in adipose tissue. Interestingly, we found that the mRNA levels of IL-1β and IL-10 were significantly elevated by IL-22 overexpression in adipose tissue (Figure 5A). The mRNA level of INF-γ was not significantly altered by IL-22 overexpression (Figure 5A). Furthermore, the mRNA levels of TNF-α and IL-6 were also significantly elevated by IL-22 overexpression (Figure 5A). These data indicate that IL-22 overexpression might induce expression of a subset of inflammatory cytokines in adipose tissue, likely contributing to the development of spontaneous liposarcomas in the mouse upon HFD feeding.

In addition, we analyzed the level of phosphorylated ERK to evaluate the activation status of Ras to ERK signaling pathway, a primary cascade involved in cell proliferation [19]. We found that phosphorylated ERK level was markedly elevated in the tumors of IL-22-TG mice (Figure 5B), indicating that Ras to ERK signaling pathway might be also implicated in the formation of spontaneous liposarcomas in IL-22-TG mice.

**Figure 6:**
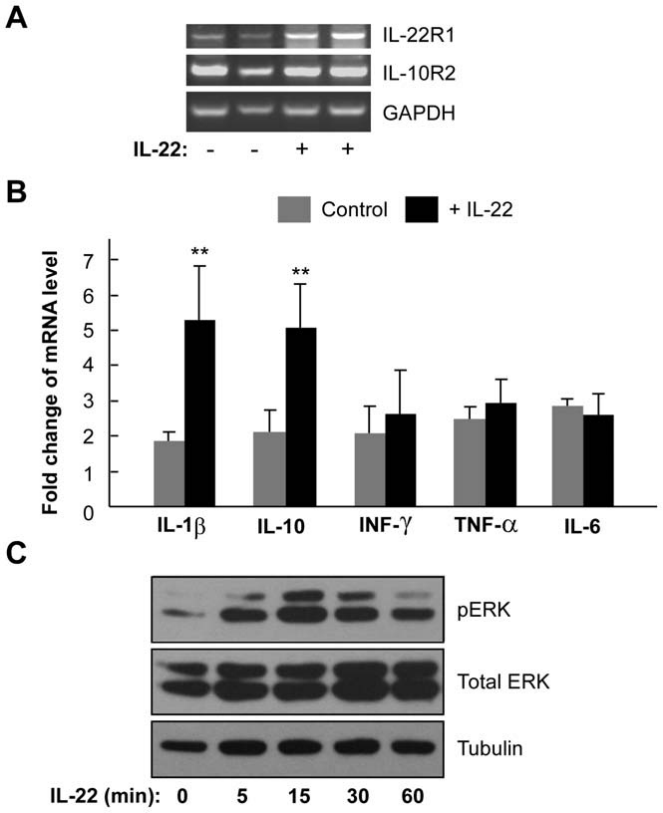
IL-22 stimulates cytokine production and ERK phosphorylation in 3T3-L1 adipocytes. (A) IL-22 receptors are expressed in differentiated 3T3-L1 cells. The mRNA levels of IL-22R1 and IL-10R2 were analyzed by RT-PCR. GAPDH was used as a loading control. The cells were treated with recombinant IL-22 (500 ng/ml) for 24 hours as indicated. (B) The expression of IL-1β and IL-10 were increased by IL-22 treatment. Differentiated 3T3-L1 cells were treated with recombinant IL-22 (500 ng/ml) for 24 hours and the total RNA was isolated and used in real-time quantitative RT-PCR to detect the expression levels of IL-1β, IL-10, INF-γ, TNF-α, and IL-6. The data are shown as mean ± SD (n = 3 for each group). ** indicates p<0.01 between the control and IL-22-treated groups. (C) Stimulation of ERK phosphorylation by IL-22 in 3T3-L1 cells. The cells were treated with recombinant IL-22 (500 ng/ml) for the length of time as indicated and the cell lysate was used in immunoblotting.

We next used a cell model to further analyze the function of IL-22 on inflammation and ERK activation in adipocytes. 3T3-L1 preadipocytes were induced to mature adipocytes using the classical “cocktail” differentiation protocol. IL-22 receptors IL-22R1 and IL-10R2 were both present in differentiated 3T3-L1 adipocytes (Figure 6A), indicating that these cells are likely responsive to IL-22. Interestingly, treatment of the cells appeared to increase the expression of IL-22R1 (Figure 6A). Similar to the findings in mouse adipose tissue (Figure 5A), IL-22 treatment was able to significantly elevate expression of IL-1β and IL-10 in differentiated 3T3-L1 adipocytes (Figure 6B). However, IL-22 had no effect on the expression of INF-γ, TNF-α and IL-6 (Figure 6B). Furthermore, IL-22 could stimulate ERK phosphorylation in a time-dependent manner (Figure 6C). Collectively, these findings indicate that IL-22-induced expression of inflammatory cytokines and activation of ERK may contribute to the formation of spontaneous liposarcomas in IL-22-TG mice fed with HFD.

## Discussion

In this study, we analyzed the potential function of IL-22 in adipose tissue. Overexpression of IL-22 in adipose tissue had no apparent effect on the development of obesity and changes of glucose homeostasis induced by high fat diet. However, high fat diet together with IL-22 overexpression could induce formation of spontaneous liposarcomas in adipose tissue with 100% penetrance. IL-22 could also induce expression of a subset of inflammatory cytokines including IL-1β and IL-10 in both adipose tissue and adipocytes. Furthermore, IL-22 could elevate the activation of ERK in both adipose tissue and adipocytes. Collectively, our study reveals for the first time that high fat diet can functionally interact with IL-22 overexpression to promote formation of spontaneous tumors in adipose tissue.

Intriguingly, high fat diet is a premise for the development of spontaneous liposarcomas in IL-22-TG mice. None of IL-22-TG mice fed with normal chow could form adipose tumors. This observation is consistent with the idea that nutritional cues are one of the major factors contributing to cancer formation [20]. It was proposed that the majority of cancers are caused by lifestyle and environmental factors, rather than gene mutations [20]. Among elements contributing to tumorigenesis, up to 35% were due to diet, and 14% to 20% were related to obesity [21]. The link between obesity and carcinogenesis is likely caused by an elevated level of chronic low-level inflammation in obesity [22]. Interestingly, it has been found that inflammation contributes to tumorigenesis [18], and that an inflammatory microenvironment is an essential component of all tumors [23]. Lately, it was found that dietary obesity was able to promote inflammation and tumorigenesis in the liver [24]. We propose here that the inflammatory responses induced by both IL-22 overexpression and obesity could jointly contribute to the development of spontaneous liposarcomas in the mouse, although the underlying molecular mechanism still awaits detailed characterization in the future.

Our study established a new liposarcoma mouse model that may facilitate liposarcoma research in the future. Currently there are very few mouse models of liposarcoma. Overexpression of the Fus-Chop fusion protein in primary mesenchymal progenitor cells is able to induce formation of myxoid liposarcoma [25]. Furthermore, the Fus-Chop fusion protein could combine with p53 deficiency to induce liposarcoma in mouse adipose-derived mesenchymal stem/stromal cells [26]. Among liposarcomas in humans, well-differentiated (WDLPS) tumors account for about 40–45% of all liposarcomas and represent the largest subgroup of adipocytic neoplasm [27]. According to the morphological and histological characteristics of liposarcoma in our model, the tumors appear to be the well-differentiated “lipoma-like” liposarcomas. In addition, we analyzed the mRNA level of MDM2 gene, a key feature of well-differentiated liposarcoma, and found that MDM2 expression was markedly elevated in the adipose tumors in IL-22-TG mice fed with HFD (data not shown). Furthermore, we found no metastasis of the tumor in these mice. These features are consistent with the findings of WDLPS in humans [28], [29].

Interestingly, both obesity and IL-22, two factors required for liposarcoma formation in the mouse, are associated with inflammatory response. We therefore propose that the altered inflammatory status by both obesity and IL-22 overexpression contribute to WDLPS tumor formation in the mouse. Consistent with this hypothesis, we did found that IL-22 is able to upregulated mRNA level of IL-1β and IL-10 in adipose tissue and adipocytes. IL-1β is a well studied cytokine with a potent pro-inflammatory effect [30]. Although IL-10 is considered to an anti-inflammatory cytokine [31], IL-10 can function as a growth factor to promote melanoma cell proliferation or possess an anti-apoptotic effect in lung cancer cells [32], [33]. In addition, we observed an increased level of ERK phosphorylation both in mouse liposarcoma samples and IL-22-treated adipocytes. This observation is consistent with the finding that IL-22 is able to activate ERK signaling pathway in other cell type [34], [35]. Among the MAPK signaling cascades implicated in tumorigenesis, ERK signaling pathway plays a vital role in protooncogene transcription and tumor cell growth [19]. Therefore, it is likely that IL-22-induced activation of ERK signaling pathway, together with the alteration of local inflammatory microenvironment impacted by both IL-22 and diet-induced obesity, may contribute to the development of spontaneous liposarcoma in the IL-22 transgenic mouse.
